# SARS-CoV-2 screening among people living in homeless shelters in Brussels, Belgium

**DOI:** 10.1371/journal.pone.0252886

**Published:** 2021-06-15

**Authors:** Michel Roland, Louisa Ben Abdelhafidh, Victoria Déom, Frank Vanbiervliet, Yves Coppieters, Judith Racapé

**Affiliations:** 1 Médecins du Monde, Brussels, Belgium; 2 Bruss’help, Brussels, Belgium; 3 Research Center in Epidemiology, Biostatistics and Clinical Research, School of Public Health, Université Libre de Bruxelles (ULB), Brussels, Belgium; 4 Chair of Health and Precarity, Faculty of Medicine, Université Libre de Bruxelles (ULB), Brussels, Belgium; Dasman Diabetes Institute, KUWAIT

## Abstract

**Background:**

Subgroups of precarious populations such as homeless people are more exposed to infection and at higher risk of developing severe forms of COVID-19 compared to the general population. Many of the recommended prevention measures, such as social distancing and self-isolation, are not feasible for a population living in shelters characterised by physical proximity and a high population density. The objective of the study was to describe SARS-CoV-2 infection prevalence in homeless shelters in Brussels (Belgium), and to identify risk factors and infection control practices associated with SARS-CoV-2 positivity rates.

**Methods:**

A total of 1994 adults were tested by quantitative PCR tests in 52 shelters in Brussels (Belgium) between April and June, 2020, in collaboration with Doctors of the World. SARS-CoV-2 prevalence is here described site by site, and we identify risk factors associated with SARS-CoV-2 positivity rates. We also investigate associations between seropositivity and reported symptoms.

**Results:**

We found an overall prevalence of 4.6% for the period, and a cluster of high rates of SARS-CoV-2 positivity (20–30% in two shelters). Among homeless people, being under 40 years of age (OR (CI95%) 2.3 (1.2–4.4), p = 0.02), having access to urgent medical care (AMU) (OR(CI95%): 2.4 (1.4–4.4)], p = 0.02), and sharing a room with someone who tested positive (OR(CI95%): 5.3 (2.9–9.9), p<0.0001) were factors associated with SARS-CoV-2 positivity rates. 93% of those who tested positive were asymptomatic.

**Conclusion:**

This study shows high rates of SARS-COV-2 infection positive tests in some shelters, with a high proportion of asymptomatic cases. The survey reveals how important testing and isolation measures are, together with actions taken by medical and social workers during the outbreak.

## Introduction

Like all crises, the coronavirus outbreak is escalating already existing social inequalities. Generally speaking, precarious populations accumulate health risks, namely lower life expectancy, more frequent co-morbidities and a more fragile mental health [1]. Moreover, they are more exposed to stress, to precarious work, and more frequently live in unsanitary and overcrowded conditions. Subgroups of precarious populations, such as homeless people, are particularly vulnerable. They are more exposed to infections and at higher risk of developing severe forms of COVID-19, compared to the general population [2]. In several U.S. cities, prevalence in homeless shelters was reported to be at 36% (in Boston), 18,5% (in Seattle), and 67% (in San Francisco), with a large proportion of asymptomatic cases [3–5] Homeless people have more physical and mental co-morbidities, which increase the risk of severe disease and mortality from SARS-CoV-2 [6, 7]. A study conducted between March 3 and May 26, 2020, at the CHU Saint-Pierre in Brussels, showed that homeless people were three times more likely to be hospitalised following a COVID-19 infection [8].

Homeless shelters are characterised by physical proximity between residents and a high population density, a lot of turnover, as well as a limited use of face masks and poor sanitary conditions. Many of the recommended prevention measures, such as social distancing and self-isolation, are not feasible for a population living in these circumstances. Support remains difficult and the people who work with them (nursing staff, social workers, volunteers) are particularly exposed. Improving the fight against COVID-19 among these populations therefore remains a challenge [9].

During the nationwide lockdown, Belgian authorities relocated symptomatic homeless people into emergency shelters and hotels. Because testing capacity was low, individuals with symptoms compatible with COVID were never tested and the actual rate of infection among the homeless in Belgium remains unknown. Mid-April, testing capacity was increased, and we were able to organise a screening campaign at homeless shelters in Brussels, Belgium, in collaboration with Doctors of the World. The objective of the study was to describe SARS-CoV-2 infection prevalence at homeless shelters in Brussels (Belgium) and to identify risk factors and infection control practices associated with SARS-CoV-2 positivity rates.

## Materials and methods

Participants were adults ≥18 years old residing in homeless shelters in Brussels. Testing occurred between April 27 and June 10, 2020, with Doctors of the World Belgium. A total of 1994 adults were tested in 52 shelters in Brussels.

### Coordination

Our testing schedule was managed using a list of Brussels shelter centres provided by Bruss’help, an organisation providing help to homeless people in Brussels [10], and responsible for coordinating emergency responses and integration policies. Priority was given to shelters with a large number of suspected cases, based on recommendations by the Belgium health institute [11]. For each testing site, a medical doctor of the testing team immediately shared the communication of results to the medical doctor working with the shelter, or directly to the patients with a positive result (in absence of a medical doctor). When a test result was positive, Doctors of the World and Bruss’help jointly coordinated communication, respectively towards the shelter medical doctors and the shelter coordination teams, in order to put in place the necessary isolation, testing and protection measures (e.g. sufficient amounts of masks and gel), as well as contact and source tracing analysis adapted to the specific context. Bruss’help organized the transfer of positive cases of shelter residents and homeless in public space to the isolation centers.

### Testing

In Belgium, the number of tests remained limited until the end of April. In the first few weeks of testing, the team performed an average of 400 PCR tests, with one or two sites visited each day. After the first week, the number of tests available increased to nearly 700 tests per week, and teams were able to test three sites per day.

All residents of each shelter were offered testing by quantitative PCR. Nasopharyngeal swabbing was done by emergency doctors trained in the appropriate nasopharyngeal swab technique.

### Data collected

At the time of testing, we collected information on demographic characteristics (age, sex), access to care (yes, Urgent Medical Care, none and unknown), previous chronic respiratory disease (yes or no), sharing a room with someone positive to SARS-CoV-2 (yes or no), viral symptoms (detailed and pooled in yes or no) and worsening of symptoms. Reported symptoms were based on the Belgium health institute recommendations [11]: fever, cold, cough/shortness of breath, loss of smell or taste, sore throat or headache.

In Belgium, people excluded from the insurance-based public health coverage are entitled to receiving free healthcare through Urgent Medical Care (AMU). Two conditions must be met: 1) that they reside in Belgium “illegally”, and 2) that their means of subsistence are below a certain threshold.

Responses were recorded electronically via a Kobo Collect form on a cell phone or tablet [12] (S1 Fig).

### Statistical analysis

We used descriptive statistics to summarise participant and shelter characteristics. We compared the proportion of positive SARS-CoV-2 tests among shelters, demographic groups, medical co-morbidities, and symptomatology using a chi2 Pearson test. Odd Ratios with their 95% Confidence Interval were reported.

The analysis was done using Stata V.16 software (StataCorp. 2017. College Station, TX).

### Ethical approval

This protocol was approved by the ethics committee of Erasme Hospital, Brussels, Belgium (Ref: P2020/431).

Doctors of the World’s Belgium-based projects comply with a charter on data collection, which states that data may be used if collected anonymously, for research or public health purposes. Consent given by patients on data confidentiality is noted in patients’ medical records. The consent was recorded orally, which was approved by the ethics committee. Due to the context of the screening campaign, language barriers, and the very low literacy rate of the beneficiaries, written consent could have difficultly been obtained. Patients may withdraw their consent at any time, or request that their files are amended or destroyed.

## Results

### Characteristics of study participants

Out of 1994 shelter residents, three people refused testing and six tests were invalid The distribution of the tests between laboratories showed that 48% of respondents were not registered in the National Population Registry. The overall case prevalence across all shelters was 4.6% (Table 1). However, prevalence was higher at the start of the campaign: in the first week, prevalence was 19,9% (56 positives to SARS-CoV-2 out of 286 tested) (Table 3). We observed a high positivity rate at three shelters, with a prevalence of 31,7%, 22.2%, and 13,8% (Table 3) during the first week of testing.

**Table 1:** Characteristics of study participants (N = 1994).

	n	%
		*mean (sd)
**Age (year)**		
^*&*^*25% missing*	1555^&^	41.9 (14.3) *
< 40	808	40.5
≥ 40	747	37.5
unknown	439	22
**Sex**		
Female	642	32.3
Male	1345	67.7
**Access to care**		
Yes	687	34.5
Urgent Medical card	584	29.3
None	615	30.8
Unknown	108	5.4
**Symptoms**		
No	1777	89.9
Yes	199	10.1
Fever	52	2.6
cold	77	3.9
Cough/ Shortness of breath	61	3.1
Loss of smell or taste	5	0.25
Sore throat or head	74	3.7
Worsening of symptoms	13/172	7.6%
**Previous chronic respiratory disease**		
No	1801	91.2
Yes	174	8.8
**Sharing a room with someone positive to SARS-CoV-2**		
No	1 573	78.9
Yes	97	4.9
Do not know	324	16.25
**PCR test**		
Negative	1894	95.4
Positive	91	4.6

32% were women and 68% were men and the mean age (standard deviation) was 41.9 (14.4). 40,5% were under 40 years old (Table 1). We have 25% missing information for this variable (mostly from the same shelter, for so-called transient migrants).

8.8% reported previous chronic illnesses, 10% had symptoms of COVID19, 5% had shared a room with someone who tested positive to SARS-CoV-2, and 14.4% did not know. Among the people for whom we obtained information, 62% had some form of access to care compared to 31% who did not (Table 1).

Regarding infection control practices, we observed a decrease in the number of positive cases to SARS-CoV-2 (2–3%) after the first week. Among those with symptoms, 7.6% worsened.

### SARS-CoV-2 risk factors

Some factors are significantly associated with SARS-CoV-2 (Table 2). People with an Urgent Medical Care card had higher proportions of SARS-CoV-2 infections compared to people devoid of access to the health system (6.5% versus 2,8%, p = 0.02);. People who shared a room with a positive person, or did not know, had significantly higher proportions of positive tests: (15, 5% and 7, % respectively versus 3,5%, p <0.0001); OR (CI 95%): 5.3 (2.9–9.9) and 2.3 (1.4–3.8) respectively.

**Table 2:** Risk factors of SARS-CoV-2.

	Negative cases n = 1894	Positive cases n = 91	N(%) positive cases	OR (CI 95%)	*p-value*
**Age (year)**	n = 1468	n = 79			
< 40	753 (51.3)	49 (62)	49 (6.1)	1	*0*.*065*
≥ 40	715 (48.7)	30 (38)	30 (4.0)	0.64 (0.40–1.03)	
**Gender**	n = 1887				
Female	612 (32.4)	29 (31.9)	29 (4.5)	1	*0*.*91*
Male	1275 (67.6)	62 (68.1)	62 (4.6)	1.03 (0.65–1.6)	
**Access to care**					* *
Yes	651 (34.4)	32 (35.2)	32 (4.7)	1	*0*.*02 *
				1.7	
Urgent Medical card	545 (28.8)	38 (41.8)	38 (6.5)	1.4 (0.87–2.3)	
No	596 (31.5)	17 (18.7)	17 (2.8)	0.58 (0.32–1.05)	
Unknown	102 (5.4)	4 (4.4)	4 (3.8)	0.80 (0.28–2.30)	
**Symptoms**	n = 1878	n = 89			
No	1685 (89.7)	83 (93.3)	83 (4.7)	1	*0*.*23*
Yes	193 (10.3)	6 (6.7)	6 (3.0)	0.63 (0.27–1.46)	
**Sharing a room with someone positive to SARS-CoV-2**					
No	1513 (79.9)	52 (57.1)	52 (3.5)	1	<0.0001
Yes	82 (4.3)	15 (16.5)	15 (15.5)	5.3 (2.9–9.9)	
Do not know	299 (15.8)	24 (26.4)	24 (7.4)	2.3 (1.4–3.8)	
**Previous chronic respiratory disease**	n = 1876	n = 90			
No	1712 (91.3)	81 (90)	81 (4.5)	1	*0*.*68*
Yes	164 (8.7)	9 (10)	9 (5.2)	1.16 (0.57–2.35)	

There were no differences in gender, previous chronic respiratory disease and symptoms between people testing positive and negative for SARS-CoV-2 (Table 2). Only 3% of people testing positive reported symptoms. There were no differences in the presence of symptoms between people testing positive and negative for SARS-CoV-2 (p = 0.23). People testing positive for SARS-CoV-2 had lower prevalence of previous chronic respiratory disease compared to people testing negative (4.5% vs 5.2%, p = 0.68). People under 40 years old were at higher risk of SARS-CoV-2 than people over 40 years old but the difference in non-significant (6.1% versus 4%, p = 0.065).

In the first week of testing, we observed clusters with SARS-CoV-2 prevalence at shelters 1 and 3, with 31.7% and 22.2% respectively, while all other shelters had very few cases. Seroprevalence was 19,6% the first week and then decreased with 1.2%, 1.7%, 4.7%, 0,4%, 0% and 0.8% the week after (Table 3).

**Table 3:** Positive cases and symptoms during testing.

	Shelters	n	Positive cases n(%)	Symptoms
**Week 1**	Shelter 1	60	19 (31,7)	1
	Shelter 2	69	8 (11.6)	2
	Shelter 3	126	28 (22,2)	1
	Shelter 4	31	1 (3.2)	0
**Week 2**	Shelter 5	5	0	
	Shelter 6	188	3 (1.6)	0
	Shelter 7	55	0	
	Shelter 8	8	0	
**Week 3**	Shelter 9	39	0	
	Shelter 10	68	1 (1.5)	0
	Shelter 11	73	1 (1.4)	0
	Shelter 12	4	0	
	Shelter 13	50	1 (2)	0
	Shelter 14	196	5 (2.6)	0
	Shelter 15	29	0	
**Week 4**	Shelter 16	8	1 (12.5)	0
	Shelter 17	4	0	
	Shelter 18	26	0	
	Shelter 19	171	3 (1.75)	0
	Shelter 20	67	2 (3)	1
	Shelter 21	19	1 (5.3)	0
	Shelter 22	172	15 (8.7)	0
**Week 5**	Shelter 23	27	0	
	Shelter 24	5	0	
	Shelter 25	12	0	
	Shelter 26	24	0	
	Shelter 27	13	0	
	Shelter 28	14	0	
	Shelter 29	14	0	
	Shelter 30	22	0	
	Shelter 31	4	0	
	Shelter 32	46	0	
	Shelter 33	14	0	
	Shelter 34	16	1 (6.25)	0
	Shelter 35	25	0	
**Week 6**	Shelter 36	11	0	
	Shelter 37	16	0	
	Shelter 38	27	0	
	Shelter 39	10	0	
	Shelter 40	8	0	
	Shelter 41	12	0	
	Shelter 42	7	0	
	Shelter 43	19	0	
	Shelter 44	15	0	
	Shelter 45	13	0	
	Shelter 46	13	0	
**Week 7**	Shelter 47	14	1 (7.1)	1
	Shelter 48	28	0	
	Shelter 49	51	0	
	Shelter 50	18	0	
	Shelter 51	9	0	
	Shelter 52	10	0	
**Total**		1985	91 (4.6%)	6 (0.7%)

Among people whotested positive, 93.3% were asymptomatic (Table 3).

## Discussion

To our knowledge, this is the first study to analyse SARS-CoV-2 seroprevalence among homeless people in Belgium. The strength of our study is its large population size, with nearly 2,000 homeless tested in Brussels. We found an overall prevalence of 4.6%. Our research has two main findings: 1/ clusters of infection during the first week of campaign testing and 2/ a high rate of asymptomatic cases.

Our study is in keeping with other studies showing high rates of asymptomatic cases and a seroprevalence that varies between shelters. In the US, prevalence levels ranged from zero to 36% in shelters [2, 3, 13]. In Paris, a recent study shows that 52% of individuals tested positive in 14 sites [14]. A study in Washington State shows a 2.0% positivity rate, but this study was carried out over 4 months of active surveillance [13]. The difference in SARS-CoV2 positivity rate may be explained by shelter characteristics and the period covered by the study.

### High infection rates clusters

Our results show a high SARS-COV-2 infection rate in the first week of testing, with important variations between sites and a prevalence of more than 30% and 20% in two homeless shelters. Prevalence decreased significantly after the first week, with very few cases in homeless shelters. This could be explained by an overall decrease of virus spread among the general population. The beginning of May shows a decline of the epidemic curve, due to lockdown [11].

Shelters presenting the highest number of positive cases have several distinct characteristics compared to other shelters: higher population density, unregistered residents, poor sanitary conditions, and insufficient protection equipment. During the first weeks of the outbreak, some shelters did not receive enough personal protection equipment; isolation centres and hotels were unavailable; and coordinators were not ready.

The observed clusters are probably related to living conditions. We have shown that sharing a bedroom with someone who tested positive is significantly associated to SARS-COV-2. Previous studies have shown that the most strongly associated seropositivity risk factors were those linked to crowded living conditions. A French survey revealed the role of collective housing in relation to viral transmission within accommodation centers [15] Another French study identified that overcrowding is the most important factor explaining variability in exposure, rather than reported adherence to recommended preventive measures [14].

A recent computer-based study of the homeless population in England also emphasised the importance of single-room accommodation, combined with heightened infection prevention methods, in preventing COVID-19 deaths [16]. Being young (<40 years) was a risk factor associated with SARS-CoV-2 detection in the homeless group, which could be due to the fact that younger homeless people are more inclined to interact socially. Probably, older people have been isolated in the center from the beginning of the outbreak.

### High rates of asymptomatic cases

The large number of asymptomatic patients is an extremely important characteristic of SARS-CoV-2 infections. Asymptomatic COVID-19 cases are characterised by the absence of symptoms but the same infectivity as symptomatic infections [17]. In our study, we observe more than 95% of asymptomatic people in some shelters. Asymptomatic patients are a source of transmission of COVID-19 in the general population and homeless shelters in particular [18]. This becomes even more important in settings with specific vulnerabilities, such as homeless shelters, where the consequences on individual and public health may be dramatic [2].

The high proportion of asymptomatic cases among shelter residents at the time of testing suggests symptom screening is insufficient to detect SARS-CoV-2 prevalence at shelters. At the time of our study, many shelter residents who had been symptomatic were already isolated, tested and housed in emergency hostels and centers, which likely led to an underestimate of SARS-CoV-2 prevalence among homeless people.

This outbreak demonstrates that waiting for detection of a symptomatic case may be too late to prevent superspreading events. Moreover, we cannot exclude that some residents may under-report symptoms for fear of losing their shelter.

A number of people were also away from the shelter during the testing period. Testing done at shelters with transient residents/ illegal migrants only reflects the residents present on the day of testing, not the entire group that intermittently uses shelter services. We know how mobile this population is, which makes tracing difficult. Some of them may fear detention if they visit a doctor or hospital, or want to protect their personal data.

Fear of isolation in the target population had a direct impact on organising the screening. Providing an upstream project evaluation time and organising an exploratory mission on site would have allowed to better understand the studied population’s needs and specificities. The urgency of the situation, and the speed at which the campaign had to be launched, did not allow for the necessary measures to be taken in order to have an in-depth analysis on the project operationalization.

### Complex homeless situations

Homeless people’s situations are complex, reflecting a mixture of service obstacles (access to health or social service) and individual barriers (language skills, mental health and trauma, mistrust of authorities, literacy levels) [19]. Service providers must focus on building rapport and trust with the residents, and take a trauma-informed approach to care, in order to persuade individuals to follow the advice [20].

The closure of regular services may put homeless people at risk of other harms, such as those associated with unsafe drug use, including acquisition of blood-borne infections such as HIV and hepatitis C. Women may also be exposed to violence. For many homeless individuals, sources of income include activities such sex work and begging, which all but disappeared. Because homeless people present a higher prevalence of health issues, social distancing is difficult and there is a fear of lockdown and isolation. Lockdown and isolation must be managed with alcohol programs, overdose prevention support and access to opioid antagonist therapies or safer supplies. Multidisciplinary teams and health promotion are essential for homeless care.

Among the 1994 homeless people, only 3 refused to be tested. This shows that, despite their living conditions, homeless populations follow public health measures, such as these screenings, if the strategy put in place is suitable for them. It is important to keep trust between workers and residents in order to have a tracing system that can be effective with illegal migrants and very precarious people.

People with Urgent Medical Care have higher proportions of SARS-CoV-2 infections compared to people without any access to healthcare. Because they have the card, we can assume that people with Urgent Medical Care are permanent residents with health issues. People without any access to healthcare are probably transient people/temporary residents who are less exposed to sheltered crowded environments and less exposed to the virus.

In terms of public health, prevention strategies are needed to minimise the cost of hospitalisation and improve healthcare access among homeless people [21]. In a modelling study of simulated adults living in homeless shelters, daily symptom screenings were associated with fewer severe acute respiratory syndrome coronavirus (SARS-CoV-2) infections and decreased costs, compared with no intervention [22].

### Limitations

This work has some limitations. We have no information on shelters’ accommodation specifics. Shelter characteristics such as population density or the capacity to maintain population stability are missing. We know that the ability and resources to implement preventative measures such as physical distancing may be crucial to prevent disease spread.

The screening campaign began too late for a true estimate of SARS-CoV-2 infection prevalence among homeless people in Brussels. The lack of testing capacities, as well as of protection equipment, undermined the work done by medical and social workers before the screening strategy was launched in the country. Emergency housing and hostels were required for homeless people during the outbreak. Testing and isolation measures, and the work done by medical and social workers during the outbreak, have been effective. Similar measures in England, designed to protect homeless people during the COVID-19 pandemic, have been effective to date [16].

Whilst we are aware of the limitations of our variables and results, collecting data on the homeless, migrants and transmigrants (who form a large part of our study) was very tricky. 48% of our population is not registered in the National Population Registry, and has no administrative and medical data. However, in the context of the COVID-19 emergency, and despite the weakness of the data, this study had an undeniable operational and public health impact. This study also highlights the need to collect more data and to improve accuracy of data on this invisible population. More frequent testing, infection control support at homeless shelters, and more permanent housing availability are clearly needed. Additional information is needed, especially on shelters’ accommodation specifics, in order to interpret our results. We also conducted a qualitative study among the homeless during the outbreak.

## Conclusion

This study shows high rates of SARS-COV-2 infection positive tests in some shelters with a high proportion of asymptomatic cases. Testing all residents at shelters, regardless of symptoms, is a strategy that has reduced transmission of SARS-CoV-2. Beyond a reduction of the risk of COVID-19 among the homeless, an increased availability of long term housing is also likely to provide health and social benefits.

## Supporting information

S1 Fig Kobo Collect form survey recorded electronically. (PDF)
