# Supplementary material for: SARS-CoV-2 screening among people living in homeless shelters in Brussels, Belgium
Source: PLoS One. 2021 Jun 15;16(6):e0252886. doi: 10.1371/journal.pone.0252886 (PMC8205130; doi:10.1371/journal.pone.0252886)
Supplement: S1 Fig — (PDF) [file pone.0252886.s001.pdf]

<u>S1 Fig:</u> Survey COVID19 Kobo Collect Form

Person's identity

Last Name *

First name *

Porte d'Ulysse number

Anonymous patient

- ◯ Yes
- ◯ No

Is the person a staff member or a resident?

- ◯ Resident
- ◯ Staff member

Gender *

- ◯ Male
- ◯ Female
- ◯ Other
- ◯ Unknown

Date of birth

yyyy-mm-dd

Access to care

- ◯ UMA/medical card
- ◯ Health insurance
- ◯ Fedasil
- ◯ European Health Insurance Card
- ◯ None
- ◯ Doesn't know

Screening

Do you agree to be tested?

◯ Yes
◯ No

Date of screening *

yyyy-mm-dd

Place of screening *

- ◯ Accueil Montfort
- ◯ Auberge DoucheFLUX
- ◯ Belgium Kitchen
- ◯ Belgium Kitchen Volxem
- ◯ Centre Ariane
- ◯ Centre Raphaël
- ◯ Chèvrefeuille
- ◯ Georges Motte
- ◯ Home 18-24
- ◯ Home Victor du Pré
- ◯ Hôtel de Bruxelles (Saint-Gilles)
- ◯ Hôtel Fierlandt (Forest)
- ◯ Hôtel Maria
- ◯ Hôtel Président (Samusocial)
- ◯ Hotel Sabina (Saint-Gilles)
- ◯ Hotel X Place St Denis (Forest)
- ◯ La Maison de la Mère et de l'Enfant
- ◯ La Parenthèse
- ◯ La Porte Ouverte
- ◯ Le Chant d'Oiseau
- ◯ Le Refuge
- ◯ Le Relais
- ◯ Les Foyers d'Accueil
- ◯ Les Petits Riens
- ◯ L'escale
- ◯ L'Ilôt
- ◯ L'Ilôt (Chaussée de Charleroi)
- ◯ Maison Rue Verte
- ◯ Molenbeek Vanderstichelen
- ◯ New Samusocial/Woluwé
- ◯ Pag-Asa
- ◯ Poverello 1
- ◯ Poverello 2
- ◯ Poverello 3
- ◯ Source
- ◯ Transit
- ◯ Trêves (Red Cross)
- ◯ Trois Pommiers

**Clinical evaluation**	**Have you recently developed any of these symptoms?**
**Cough, dyspnea, chest pain**	◯ Yes ◯ No

**Anosmia (loss of sense of smell), dysgeusia (taste problem)**	◯ Yes ◯ No
**Fever**	◯ Yes ◯ No
**Muscle pains**	◯ Yes ◯ No

**Fatigue**	◯ Yes ◯ No
**Rhinitis**	◯ Yes ◯ No
**Sore throat**	◯ Yes ◯ No
**Headaches**	◯ Yes ◯ No
**Anorexia**	◯ Yes ◯ No
**Aqueous diarrhea without apparent cause**	◯ Yes ◯ No

**Acute confusion**	◯ Yes ◯ No
**Sudden fall without apparent cause**	◯ Yes ◯ No

Are you suffering from a chronic respiratory disease (COPD, asthma,...)?

◯ Yes
◯ No

Have the symptoms worsened?

◯ Yes
◯ No

Have you shared a room with a person who has been tested positive to COVID19?

- ◯ Yes
- ◯ No
- ◯ Doesn't know

DO NOT MODIFY THIS CODE. WRITE THIS CODE ON THE CARD PROVIDED AND GIVE IT TO THE PERSON. ALSO GIVE THE CODE TO THE DOCTOR AND THE STAFF AT THE CENTRE.

The person's code is :

Results

TO BE COMPLETED AFTER RECEIPT OF RESULTS

Date of receipt of results

yyyy-mm-dd

Screening results

- ◯ Positive
- ◯ Negative
- ◯ Invalid
